# A Fluorescent Oligothiophene-Bis-Triazine ligand interacts with PrP fibrils and detects SDS-resistant oligomers in human prion diseases

**DOI:** 10.1186/s13024-016-0074-7

**Published:** 2016-01-26

**Authors:** Thibaut Imberdis, Adeline Ayrolles-Torro, Alysson Duarte Rodrigues, Joan Torrent, Maria Teresa Alvarez-Martinez, Gabor G. Kovacs, Jean-Michel Verdier, Mike Robitzer, Véronique Perrier

**Affiliations:** Université Montpellier, Montpellier, F-34095 France; Inserm, U1198, Montpellier, F-34095 France; EPHE, Paris, F-75007 France; Etablissement Confiné d’Expérimentation ECE, CECEMA, US009 Biocampus, UMS 3426, Université Montpellier, Montpellier, F-34095 France; Institute of Neurology, Medical University Vienna, A-1097 Vienna, Austria; Institut Charles Gerhardt Montpellier, UMR 5253 CNRS-UM2-ENSCM-UM, Matériaux Avancés pour la Catalyse et la Santé, ENSCM, 8 rue de l’Ecole Normale, 34296 Montpellier cedex 5, France

**Keywords:** Neurodegenerative diseases, Prions, Fluorescent oligothiophene ligand, SDS-resistant oligomers, Diagnosis

## Abstract

**Background:**

Prion diseases are characterized by the accumulation in the central nervous system of an abnormally folded isoform of the prion protein, named PrP^Sc^. Aggregation of PrP^Sc^ into oligomers and fibrils is critically involved in the pathogenesis of prion diseases. Oligomers are supposed to be the key neurotoxic agents in prion disease, so modulation of prion aggregation pathways with small molecules can be a valuable strategy for studying prion pathogenicity and for developing new diagnostic and therapeutic approaches. We previously identified thienyl pyrimidine compounds that induce SDS-resistant PrP^Sc^ (rSDS-PrP^Sc^) oligomers in prion-infected samples.

**Results:**

Due to the low effective doses of the thienyl pyrimidine hits, we synthesized a quaterthiophene-bis-triazine compound, called MR100 to better evaluate their diagnostic and therapeutic potentials. This molecule exhibits a powerful activity inducing rSDS-PrP^Sc^ oligomers at nanomolar concentrations in prion-infected cells. Fluorescence interaction studies of MR100 with mouse PrP fibrils showed substantial modification of the spectrum, and the interaction was confirmed *in vitro* by production of rSDS-oligomer species upon incubation of MR100 with fibrils in SDS-PAGE gel. We further explored whether MR100 compound has a potential to be used in the diagnosis of prion diseases. Our results showed that: (*i*) MR100 can detect rSDS-oligomers in prion-infected brain homogenates of various species, including human samples from CJD patients; (*ii*) A protocol, called “Rapid Centrifugation Assay” (RCA), was developed based on MR100 property of inducing rSDS-PrP^Sc^ oligomers only in prion-infected samples, and avoiding the protease digestion step. RCA allows the detection of both PK-sensitive and PK-resistant PrP^Sc^ species in rodents samples but also from patients with different CJD forms (sporadic and new variant); (*iii*) A correlation could be established between the amount of rSDS-PrP^Sc^ oligomers revealed by MR100 and the duration of the symptomatic phase of the disease in CJD patients; and (*iv*) Bioassay experiments showed that MR100 can trap prion infectivity more efficiently than P30 drug.

**Conclusions:**

MR100 is a powerful tool not only for studying the prion aggregation pathways regarding oligomeric and sPrP^Sc^ species, but also for developing alternative methods for the detection of prion-infected samples. Considering our bioassay results, MR100 is a promising molecule for the development of prion decontamination approaches.

**Electronic supplementary material:**

The online version of this article (doi:10.1186/s13024-016-0074-7) contains supplementary material, which is available to authorized users.

## Background

Prions are unconventional infectious agents responsible for fatal neurodegenerative disorders in animals and humans [1]. In humans, prion diseases are mostly represented by Creutzfeldt-Jakob disease (CJD) which is classified into three groups: sporadic (85 % of cases), genetic (10-15 %) and acquired (less than 5 %) [2]. The disease is characterized by abnormal prion protein (PrP^Sc^) deposits in the brain of CJD patients, often forming large amyloid plaques and fibrils. The crucial step in the transmission and manifestation of prion diseases is the conversion of benign monomeric cellular prion proteins (PrP^C^) into pathogenic multimeric PrP^Sc^ isoforms [3, 4]. Structural differences confer specific biochemical properties: PrP^C^ is detergent-soluble and completely digested by proteinase K (PK), whereas PrP^Sc^ is detergent-insoluble, tends to oligomerize to form fibrils and is partially resistant to PK. PrP^Sc^ digestion by PK produces a trimmed C-terminal fragment, called PrP(27-30) or PK-resistant PrP^Sc^ (rPrP^Sc^), and is considered to be the main biomarker of prion disease. The proteinase K digestion assay performed on brain tissue is the referenced method used for the diagnosis of human prion diseases. However, a growing number of evidences shows that: (*i*) A novel prion disease described by Gambetti in 2008, demonstrated that 2-3 % of CJD cases are characterized by a prion protein highly sensitive to proteinase K digestion and difficult to detect in classical conditions. In the absence of PK-resistant marker (because those cases do not have mutations in the PRNP gene) they can be easily missed as prion cases [5–8]; (*ii*) PK-sensitive PrP^Sc^ fraction (sPrP^Sc^) represents more than 80 % of the total PrP^Sc^ isoforms, thus it could be interesting to find methods targeting these species to better understand their role [9–12]; and (*iii*) the crucial role of oligomeric species recognized as the most toxic forms, not only in prion diseases but also for most prionopathies, whereas amyloid fibrils have a role in sequestering dangerous soluble oligomers should be taken into account when searching for anti-amyloid drugs [13–15]. Altogether, these studies underline the necessity of finding new tools to study the various PrP^Sc^ species and their role in prion pathogenicity for developing new diagnostic and therapeutic strategies. Recently, by using virtual and cellular drug screenings, we identified a family of thienyl pyrimidine drugs (P30 and A6 are the lead compounds among 50 molecules tested) that allow us to detect SDS-resistant PrP^Sc^ (rSDS-PrP^Sc^) dimers and trimers after proteinase K digestion [16]. These rSDS-PrP^Sc^ oligomers are only observed in prion-infected samples, suggesting a potential use for prion diagnosis. The low affinity of P30 for the normal PrP^C^ isoform was confirmed by Surface Plasmon Resonance studies (SPR or Biacore) with a calculated affinity of 147 ± 50 uM. The binding of P30 for PrP^Sc^ could not be evaluated because of aggregation issues in the Biacore sensor chip. Other biochemical studies showed that: (*i*) P30 did not cross-link recombinant full-length PrP (recPrP) or fibrils *in vitro*; (*ii*) P30 did not modify the kinetics of conversion of recPrP into amyloid fibrils in a semi-automated assay; and (*iii*) The effective doses (ED_50_: concentration at which 50 % of rSDS-PrP^Sc^ oligomers was achieved) of P30 and A6 were in the micromolar range, which is too low to understand their mechanism of action *in vitro*. Indeed, it was not possible to decipher if P30: (*i*) catalyzes oligomers from PrP^Sc^ monomers, or (*ii*) cross-links pre-existing small oligomers as dimers and trimers, or (*iii*) interacts with larger aggregates such as fibrils, and under denaturing conditions and migration on SDS-PAGE gel, rSDS-PrP^Sc^ dimers and trimers are observed.

In order to better understand the mechanism of action of previously identified thienyl pyrimidine compounds [16], as well as to evaluate their diagnostic and therapeutic potentials, we searched for analogs with stronger activity. Based on the results of a structure-activity study, we synthesized a quaterthiophene-bis-triazine compound, called MR100 [17, 18]. Our main results show that: (*i*) MR100 exhibits a very powerful rSDS-PrP^Sc^ oligomer-inducing activity at nanomolar concentrations in prion-infected cells; (*ii*) fluorescence interaction studies of MR100 with mouse PrP fibrils showed substantial modification of the spectrum, suggesting the binding of MR100 to PrP fibrils; (*iii*) the *in vitro* binding was confirmed in SDS-PAGE gels, showing production of rSDS-oligomer species upon incubation of MR100 with fibrils, and the rSDS-oligomeric species were colored orange as is MR100; (*iv*) MR100 can detect rSDS-oligomers in prion-infected brain homogenates of various species; (*v*) a protocol, called “Rapid Centrifugation Assay” (RCA), based on MR100 property of inducing rSDS-PrP^Sc^ oligomers only in prion-infected samples, and avoiding the protease digestion step was developed. RCA allows the detection of both PK-sensitive and PK-resistant PrP^Sc^ species in prion-infected samples from rodents and also from patients with different CJD forms (sporadic and new variant); (*vi*) a correlation was established between the amount of rSDS-PrP^Sc^ oligomers revealed by MR100 and the duration of the symptomatic phase of the disease in CJD patients; and (*vii*) bioassay experiments show that MR100 can trap prion infectivity more efficiently than P30 drug, suggesting a potential of MR100 as a surface prion decontaminant.

## Results

### Design and synthesis of MR100, a derivative that combines the chemical characteristics of the thienyl pyrimidine compounds A6 and A18

We previously identified thienyl pyrimidine compounds that allow the detection of rSDS-PrP^Sc^ dimers and trimers on western blot [16]. A structure-activity study performed on 23 derivatives of P30 showed that the activity depended on the presence of a donor group of π electrons (NH- or SH-) branched to a conjugated C6 heterocycle, such as a pyrimidine or an azine, and of an acceptor group of π electrons (Br-, Cl-, CN-) branched to a thienyl cycle (Table 1) [16, 17]. We also found that the addition of a second thienyl cycle (such as the A6 and A14 compounds), instead of a halogen atom, accentuated their electro-attractive character and increased the rSDS-PrP^Sc^ oligomer-inducing activity [17]. We hypothesized that dimerization of the thienyl pyrimidine scaffold of A6 could enhance delocalization of π electrons and further increase its rSDS-PrP^Sc^ oligomer-inducing activity. We thus synthesized MR100, a bivalent ligand with a chemical scaffold that is similar to an A6 dimer and includes the triazine cycle of A18 (Table 1) [18].

**Table 1:**
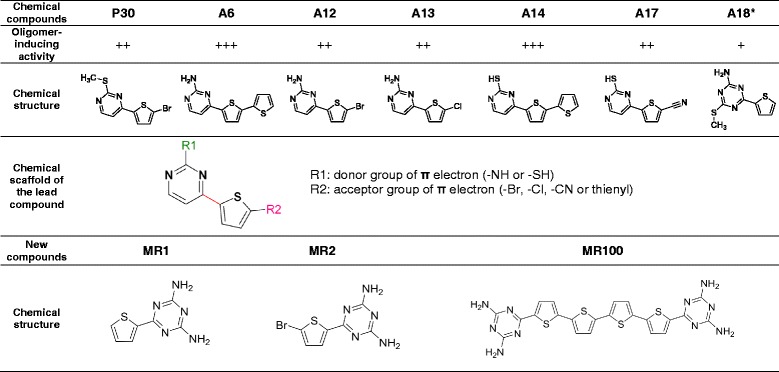
Chemical structure of thienyl pyrimidine compounds with oligomer-inducing activity and schematic representation of the synthesis of the new derivative, MR100

### MR100 rSDS-PrP^Sc^ oligomer-inducing activity is five thousand times stronger than that of A6

**Fig. 1:**
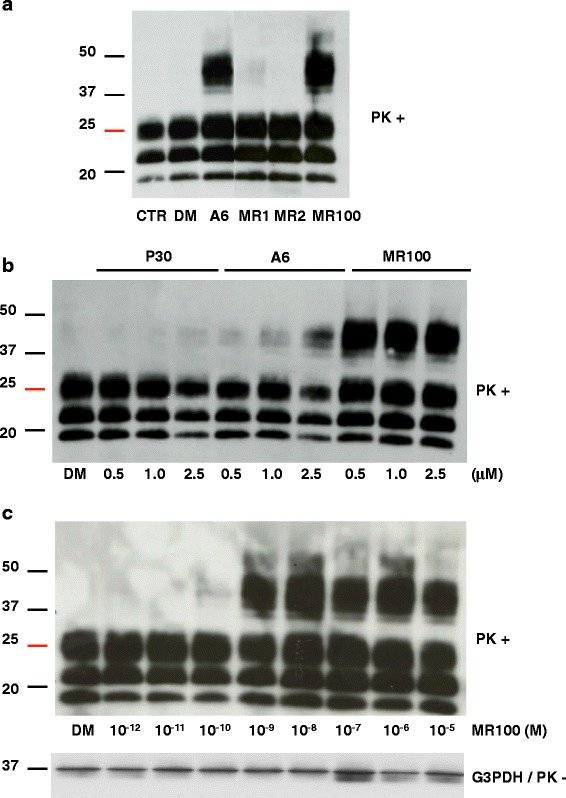
MR100 has a stronger PrP^Sc^ oligomer-inducing activity in prion-infected N2a58/22 L cells than P30 and A6. **a** Effect of the newly synthesized MR1, MR2 and MR100 compounds in prion-infected N2a58/22 L cells. Cells were left untreated (CTR) or incubated with 20 μM of A6 (positive control), MR1 and MR2 (synthesis intermediates), MR100, or 20 μL DMSO (DM) for 4 days. Protein lysates were analyzed by immunoblotting with the SAF mix (a mixture of the anti-PrP SAF60, SAF69 and SAF70 monoclonal antibodies) after proteinase K (PK) digestion. **b** Comparison of the oligomer-inducing activity of P30, A6 and MR100. Prion-infected N2a58/22 L cells were incubated with 0.5, 1 or 2.5 μM of each compound, or 20 μL DMSO (DM) for 4 days. Protein lysates were then analyzed by immunoblotting with the SAF mix after PK digestion. **c** MR100 dose-response curve in prion-infected N2a58/22 L cells. Successive dilutions of MR100 in DMSO were used to obtain final concentrations ranging from 10^-12^M (1pM) to 10^-5^M (10 μM). Cells were incubated for 4 days and at confluence they were lysed. Protein lysates were analyzed by immunoblotting with the SAF mix after PK digestion according to the previously described protocol [16, 30]. Loading control was performed with antibodies against glyceraldehyde-3-P dehydrogenase (G3PDH) and before proteinase K digestion. Molecular masses (20–50 kDa) are indicated on the left side of the panels

We then tested whether MR100 and the two synthesis intermediates MR1 and MR2 could allow the detection of rSDS-PrP^Sc^ dimers and trimers in prion-infected mouse neuroblastoma N2a58/22 L cells. N2a58/22 L cells were incubated with 20 μM of each compound or with A6, used as a positive control, for 4 days. Negative controls were untreated cells (CTR), or incubated with vehicle (DMSO) alone. When cells reached confluence, they were lysed, PK-digested and analyzed by immunoblotting with the SAF mix of anti-PrP antibodies. MR100 had a strong rSDS-PrP^Sc^ oligomer-inducing activity, whereas only traces were detected in cells incubated with MR1 and no rSDS-PrP^Sc^ oligomer was visible in samples incubated with MR2 (Fig. 1a).

Because the MR100 compound seemed to be more potent than A6, we performed dose-response curves using a range of concentrations (from 0.5 to 2.5 μM) of MR100, P30 and A6 (Fig. 1b). rSDS-PrP^Sc^ oligomers could not be detected when P30 was used at such low concentrations because its ED_50_ was previously estimated at 17 μM. Incubation of N2a58/22 L cells with 2.5 μM A6 (its estimated ED_50_) [16] induced moderate rSDS-PrP^Sc^ oligomer formation. Conversely, the lowest tested concentration (0.5 μM) of MR100 was already sufficient to saturate the signal, indicating that MR100 is more effective than P30 and A6 (Fig. 1b). To determine the ED_50_ of MR100, we performed a dose-response curve in N2a58/22 L cells with MR100 concentrations ranging from 1 pM (10^-12^ M) to 10 μM (10^-5^ M). Oligomers were detected when only 0.1 nM MR100 was used and the signal was saturated with 1nM MR100, suggesting that MR100 ED_50_ was between 0.1 and 1 nM (Fig. 1c). Therefore, we estimated that MR100 is active at concentrations 5000 times lower than A6 and 30,000 times lower than P30.

### MR100 does not oligomerize PrP^C^ in either normal or prion-infected cells

**Fig. 2:**
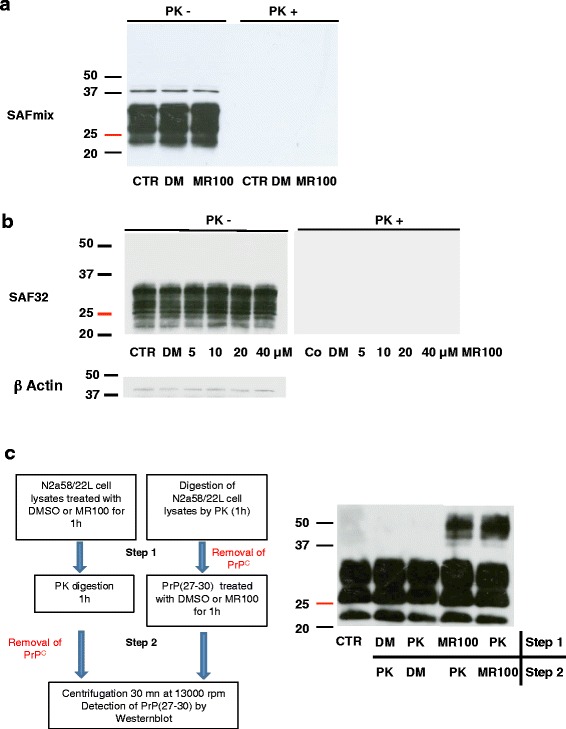
MR100 did not induce SDS resistant PrP^C^ oligomers. **a** Parental non-infected cells, N2a58, were left untreated (CTR) or incubated with 20 μM of MR100 or 20 μL DMSO (DM) for 4 days. Protein lysates were analyzed by immunoblotting with the SAF mix before or after PK digestion. **b** N2a58 cell lines were left untreated (CTR) or incubated with various concentration of MR100 from 5 to 40 μM or 40 μL of DMSO (DM) for 4 days. Protein lysates were analyzed by immunoblotting with the SAF 32 before or after PK digestion. Loading control was performed with antibodies against β actin and before proteinase K digestion. **c** Schematic representation of the protocols used to test if PrP^C^ isoforms are part of rSDS-oligomers (Left panel). First step, N2a58/22 L lysates were incubated with 20 μM of MR100 or with proteinase K to eliminate PrP^C^, then in the second step, MR100-exposed lysates were digested with proteinase K, while proteinase K digested samples were incubated with MR100. PrP^Sc^ species were then analyzed by western blotting. Western blot analysis of the samples processed according to the two different protocols using the SAFmix of anti-PrP antibodies (Right panel). CTR, untreated samples, digested by proteinase K; DM, DMSO. Molecular masses (20–50 kDa) are indicated on the left side of the panels

Since MR100 is more potent than P30 and A6, we wanted to determine whether the new drug could also induce the oligomer-induced activity on PrP^C^ isoforms. To this aim, N2a58 cells corresponding to the parental non-infected line, were first incubated with 20 μM of MR100 for 4 days. Western blot analysis of the cell lysates was performed using SAFmix, since it recognizes both PrP^C^ and PrP^Sc^ isoforms and labels the rSDS-PrP^Sc^ oligomers very strongly. We hypothesized that if rSDS-PrP^C^ oligomers exist, SAFmix should also recognize these species. However, western blot analysis did not show rSDS-PrP^C^ oligomers in N2a58 cells upon treatment with MR100 either before or after PK digestion (Fig. 2a). We also used SAF32, whose epitope recognizes the octarepeats (epitopes 59-65) in the N-terminal region of the protein, since normal cells have a higher proportion of full-length PrP^C^ protein than truncated protein. We did not observe any rSDS-PrP^C^ oligomers using SAF32 in non-infected cells treated with a range of MR100 concentration (Fig. 2b). These results are in accordance with our previous data using P30 and A6 [16].

Our second hypothesis was that MR100 oligomerizes PrP^C^, via PrP^Sc^ in prion-infected N2a58/22 L samples. Indeed, MR100 could play the role of catalyst for the conversion of PrP^C^ to PrP^Sc^, as a template for the folding of PrP^C^, leading to subsequent oligomerization. To decipher this, we performed 2 experiments in parallel on freshly made prion-infected cellular lysates. First, prion-infected cell lysates were either treated with MR100 (or DMSO as a negative control) or with PK, after which the samples previously treated with MR100 or DMSO were PK-digested, and those previously PK-digested were treated with MR100 or DMSO (see schema Fig. 2c). We expected that, if PrP^C^ is implicated in the process of oligomerization, the samples that were first PK treated in order to remove PrP^C^ would have fewer oligomers than samples incubated first with MR100. This was not the case: samples that were first digested with PK to remove PrP^C^ before the incubation with MR100 did not exhibit fewer oligomers than samples incubated first with MR100 and then PK digested as shown in the western blot (Fig. 2c). This result suggests that PrP^C^ is not implicated in the process of oligomerization.

### Fluorescent studies showed that MR100 interacts with recPrP proteins

**Fig. 3:**
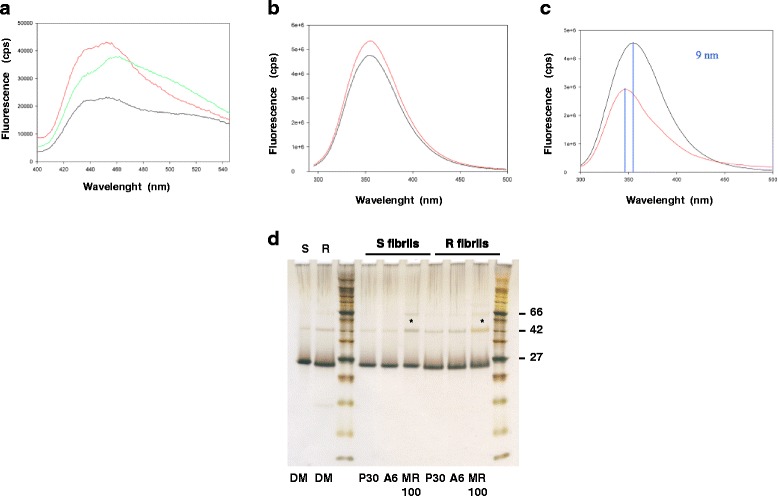
Fluorescence interaction studies between MR100 and PrP. **a** Fluorescence studies of MR100 compound incubated with purified recombinant MoPrP(23-230) proteins. 4 μM of MoPrP(23-230), either soluble or fibrillar, were incubated with 50 μM of MR100 in 1 % DMSO, 50 mM MES buffer pH6, during 2 h at 25 °C. Emission spectra between 400 and 550 nm were recorded by exciting at 470 nm: 50 μM of MR100 (black), 50 μM of MR100 + α-soluble MoPrP(23-230) (red), 50 μM of MR100 + fibrils of MoPrP(23-230) (green). **b**-**c** Fluorescence of tryptophan and tyrosine residues of soluble MoPrP(23-230) alone (black), or incubated with 1 % DMSO, 50 mM MES buffer pH6, during 2 h at 37 °C (red) (**b**). Fluorescence of tryptophan and tyrosine residues of soluble MoPrP(23-230) alone (black), or incubated with 50 μM of MR100 in 1 % DMSO, 50 mM MES buffer pH 6, during 2 h at 37 °C (red) (**c**). All spectra were recorded at 290 nm. **d** Hamster-S or -R fibrils at a concentration of 4 μM were incubated with solvent alone (1 % DMSO, 50 mM MES buffer pH6), or 40 μM of P30, A6, or MR100 compounds in 1 % DMSO, 50 mM MES buffer pH6, during 2 h at 25 °C. Samples were then mixed with loading buffer, boiled for 15 min at 90 °C and loaded on a 12 % SDS-PAGE gel. Proteins in the gel were revealed by silver staining. Molecular weight markers indicated: 27, 42 and 66 kDa. Asterisks showed dimer and trimer bands that are increased following incubation with MR100 compound

Interaction studies were performed to check whether the MR100 compound binds to prion protein. We used purified recombinant full-length mouse prion protein MoPrP(23-230) folded into α-helices or fibrils to perform Surface Plasmon Resonance (SPR or Biacore) studies, as previously performed for P30 and A6 compounds [16]. Unfortunately, the experimental conditions to measure the interaction between MR100 and MoPrP(23-230) failed because we had to dilute the MR100 in buffer conditions that render it too insoluble to determine affinity measurements. Since MR100 was an orange-colored compound containing several heterocycles, such as thienyl and azine, some potential fluorescence properties could be used for interaction studies. Absorption spectra were recorded for MR100 from 200-600 nm and the maximal absorption wavelength of the compound was detected at 470 nm and was thus selected for excitation of the molecule with a fluorimeter. After excitation of the MR100 compound at 470 nm, the maximal emission wavelength of fluorescence was recorded at 531 nm. We took advantage of these physical properties to perform interaction studies between MR100 and recombinant PrP proteins, by following the fluorescence of MR100 in presence or in absence of MoPrP(23-230) after 2 h of incubation. Emission spectra were recorded at an excitation wavelength of 470 nm and showed that, in presence of the soluble MoPrP(23-230), the maximal fluorescence signal at 452 nm is doubled compared to control MR100 alone (Fig. 3a). For PrP fibrils, we also observed an increase of the fluorescence signal but also a strong red-shift of the emission spectrum: the maximal wavelength shifted from 452 nm to 460 nm, and a large shoulder appeared around 500 nm (Fig. 3a). These results illustrated significant modifications in the environment of the MR100 compound in presence of PrP proteins and showed that MR100 molecules interact with both isoforms of MoPrP(23-230). The strong red-shift observed for the PrP fibrils may be explained by a specific interaction of MR100 with the quaternary structural elements of the fibrils. As a control, we followed the fluorescence of the aromatic residues (Tyr, Trp) of soluble MoPrP(23-230) at 295 nm, before the incubation with the solvent (DMSO) or MR100 compound and after 2 h of incubation. For MoPrP(23-230) incubated with DMSO, spectra recorded before addition of the solvent and after 2 h of incubation are very similar indicating that the solvent did not significantly modify the fluorescence properties of the protein (Fig. 3b). However, when MoPrP(23-230) is incubated with MR100, the spectra recorded before addition of MR100 and after 2 h of incubation with the molecules are substantially different (Fig. 3c) as we observed a decrease of the protein fluorescence signal by 50 % and a blue-shift of the maximal wavelength by 9 nm. Upon binding with MR100, a quenching of the fluorescence of the aromatic residues of the recPrP is observed due to a more hydrophobic environment that is also illustrated by a blue-shift from 355 to 346 nm. These results indicate that the environment of the aromatic residues of the prion protein is modified in presence of MR100 and confirm the binding between MR100 and MoPrP(23-230) previously observed by MR100 fluorescence. Alternatively, we performed a fluorescence binding experiments using Hamster S- or R-fibrils, produced by different agitation modes (shaking or rotated) and displaying distinctive morphologies (R-fibrils are curved, S-fibrils are straight) linked to different folding patterns of cross–β structures [19, 20]. Our objective was to see whether MR100 could preferentially bind to Hamster S- or R-fibrils. Using the fluorescent property of MR100 as a tracer, we showed that MR100 interacts with both types of fibrils although the intensity of the fluorescence is higher with R-fibrils than S-fibrils (data not shown). After 2 h of incubation in DMSO, P30, A6 or MR100, with hamster S- or R-fibrils, each mixture was loaded on a SDS-PAGE gel to directly visualize binding by inducing more rSDS-oligomers. Upon incubation of hamster fibrils with MR100 compound we could see an increase of rSDS-dimers and trimers for both S- and R-fibrils compared to controls incubated with DMSO (Fig. 3d). Remarkably, for hamster R-fibrils, the bands corresponding to rSDS-dimers and trimers appeared orange, as did the MR100 compound, indicating a direct interaction between MR100 and PrP. We could not directly visualize the interaction between P30 and A6 compound likely because the affinity of P30 and A6 compounds for PrP is lower, as suggested by their ED_50_.

### MR100 allows rSDS-PrP^Sc^ oligomer detection in brain homogenates infected by different rodent strains

**Fig. 4:**
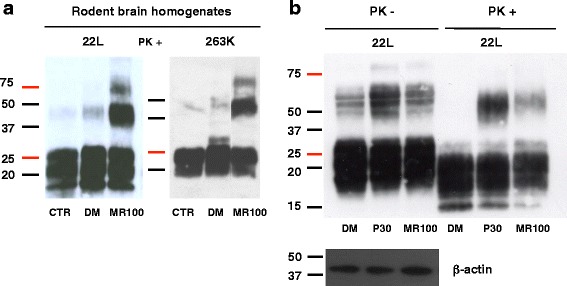
MR100 shows oligomer-inducing activity in brain homogenates from prion-infected rodents. **a** MR100 oligomer-inducing activity was tested using freshly homogenized rodent brain tissues infected with the 22 L (mice) or the 263 K prion strain (hamsters). Fifty μL of 10 % mouse or hamster brain homogenates were diluted in 300 μL PBS/2 % Sarkosyl, incubated with 1.5 mM MR100 (corresponding to 150 μL of 5 mM MR100) at room temperature for 1 h or with 150 μL of DMSO as control, and then digested with 20 μg/ml PK at a ratio of 1:50 (PK/proteins). PK digestion was stopped by addition of a cocktail of protease inhibitors (Complete), before analysis of rPrP^Sc^ by western blotting with the SAF mix according to the previously described protocol [16]. CTR: untreated 22 L- or 263 K-infected brain homogenates (negative control); DM: 22 L- or 263 K-infected brain homogenates incubated with DMSO. **b** Comparison of P30 and MR100 oligomer-inducing activity on the 22 L prion strain, before and after proteinase K digestion. Fifty μL of 10 % 22 L-infected brain homogenates were diluted in 350 μL PBS/2 % Sarkosyl, incubated with 1 mM MR100 or P30 (corresponding to 100 μL of 5 mM MR100 or P30), at room temperature for 1 h. Then, aliquots of 30 μL were taken before addition of proteinase K, to perform western blot (PK-) probed with SAF mix, but also with anti-β-actin antibodies as loading controls. The rest of the sample was then digested with 20 μg/ml PK at a ratio of 1:50 (PK/proteins) (PK+). The reaction was stopped by addition of the protease inhibitor cocktail, before analysis of rPrP^Sc^ by western blotting with the SAF mix as in A. DM: 22 L-infected brain homogenates incubated with 100 μL DMSO. Molecular masses (20–50 kDa) are indicated on the left side of the panels

We previously showed that P30 can induce rSDS-PrP^Sc^ oligomers in brain homogenates of mice infected with the scrapie strain 22 L, but not in non-infected samples [16]. To assess whether MR100 also had this activity *in vitro*, freshly homogenized 22 L-infected mouse or 263 K-infected hamster brain homogenates were incubated with 1 mM MR100 at room temperature for 1 h, then PK-digested and the reaction stopped by addition of a protease inhibitor cocktail. Negative controls were untreated samples (CTR), or 22 L- or 263 K-infected rodent brain homogenates incubated with DMSO alone (DM). Immunoblotting with the SAFmix showed a strong signal of rSDS-PrP^Sc^ dimers and trimers mostly in the samples incubated with MR100 (Fig. 4a), suggesting that the MR100 mechanism of action is similar to that of P30 and A6. The oligomer signal observed with DMSO after proteinase K digestion is much lower than the one observed in brain homogenates incubated with the MR100 compound (Fig. 4a). To assess the extent of proteinase K resistance of these rSDS-PrP^Sc^ species, a western blot of 22 L-infected brain homogenate incubated with DMSO, P30 or MR100, was made before and after proteinase K digestion. The results clearly showed the presence of many more oligomers before PK than after PK+, for all conditions (DMSO, P30 and MR100), illustrating that these oligomers are indeed PK sensitive. The DMSO control also reveals the presence of low amounts of SDS-resistant oligomers before PK that are not resistant to the digestion by PK, as shown in Fig. 4b. In addition, we noticed that when brain homogenates are treated with 1 mM of either P30 or MR100, the amount of SDS-resistant oligomers is lower for MR100 than for P30, by western blot. This lower signal is likely due to a strong precipitation effect that we visually observed with MR100 compound and not with P30.

### The “rapid centrifugation assay” allows the detection of infected samples based on rSDSPrP^Sc^ oligomer formation induced by MR100

**Fig. 5:**
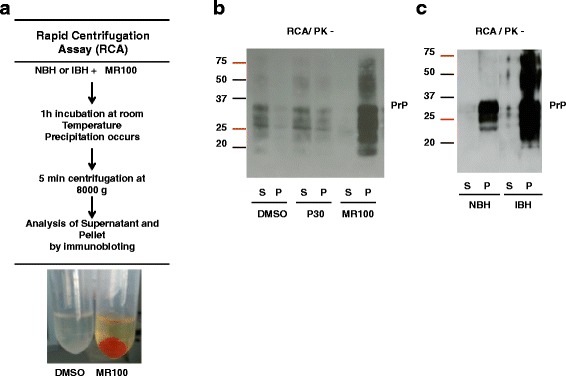
A MR100-based assay can differentiate between prion-infected and normal brain homogenates without proteinase K digestion. **a** Schematic description of the RCA protocol to test brain homogenates without PK digestion. Brain tissues were freshly homogenized in microbead-containing tubes. Normal brain homogenates (NBH) or prion-infected brain homogenates (IBH) were incubated with MR100 for 1 h, at room temperature, leading to a precipitation of PrP isoforms. After a short centrifugation step, the pellet with MR100 (orange tube) concentrates PrP isoforms, whereas no pellet is detectable with DMSO. **b** Comparison of DMSO, P30 and MR100 precipitation capabilities using the RCA protocol. Fifty μL of 10 % 22 L infected brain homogenates were diluted in 300 μL of PBS/2 % sarcosyl and incubated using either 1.5 mM of P30 or MR100 or an equivalent volume of the solvent alone (DMSO), at room temperature for 1 h. Then, samples were centrifuged at 8000 g for 5 min. Supernatants (S) were collected and 30 μL of each supernatant was mixed with an equivalent volume of 2X loading buffer. Pellets (P) were resuspended in 30 μL PBS/2 % Sarcosyl, and mixed with an equal volume of 2X loading buffer. Thirty microliters of each sample were loaded on 12 % Bis-Tris gels (Criterion, Biorad) and immunoblotting was carried out with the SAF mix according to standard procedures [16]. The samples (S/P) were analyzed by western blotting using SAF mix anti-PrP antibodies. **c** Comparison of infected versus non-infected brain homogenates processed with the RCA protocol. Fifty microliters of 10 % freshly homogenized brain tissues from normal (NBH) or 22 L prion-infected (IBH) mice were processed according to the RCA protocol described in A and B. Thirty microliters of supernatant (S) or pellet (P) were loaded on 12 % Bis-Tris gels (Criterion, Biorad) and immunoblotting was carried out with the SAF mix as described above. Molecular masses (20–75 kDa) are indicated on the left side of the panels

**Fig. 6:**
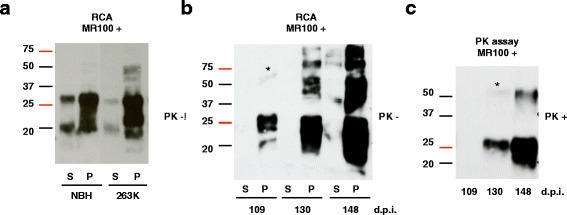
RCA can detect rSDS-PrP^Sc^ oligomers at early stages of the disease. **a** Fifty microliters of 10 % hamster brain homogenates (NBH or infected with the 263 K prion strain) were diluted in 300 μL of PBS/2 % Sarkosyl, incubated with 1.5 mM MR100 at room temperature for 1 h and then centrifuged at 8000 g for 5 min. Supernatants (S) were collected and 30 μL of each supernatant were mixed with an equivalent volume of 2X loading buffer. Pellets (P) were resuspended in 30 μL PBS/2 % Sarcosyl, and mixed with 30 μL of 2X loading buffer. Thirty microliters of each sample were loaded on 12 % Bis-tris gels (Criterion, Biorad) and western blotting was carried out with the SAF mix according to standard procedures [16]. Molecular masses (20–75 kDa) are indicated on the left side of the panels. **b** Hamster brain tissues were collected at various days post-infection (d.p.i.), as indicated, and freshly homogenized tissues were processed according to the RCA protocol using MR100 and analyzed by immunoblotting as described above. **c** To compare the RCA and the PK test, the same hamster brain homogenates (at 109, 130 and 148 d.p.i.) were incubated with MR100 at room temperature for 1 h, then digested with 20 μg/mL of proteinase K and processed as described in the legend to Fig. 4a. The asterisk in **b** and **c** indicates the position of oligomer traces

To determine whether prions were indeed precipitated following incubation with high concentrations of MR100, we developed a new protocol in which, following MR100 incubation, brain homogenates were separated in pellet and supernatant by quick centrifugation and immediately analyzed by immunoblotting without PK digestion. We first observed an orange-colored precipitate in the tube containing MR100, whereas no pellet was visible for samples treated with DMSO (Fig. 5a). By using the “rapid centrifugation assay” (RCA) we evaluated the ability of the various molecules (DMSO, P30, MR100) to precipitate PrP isoforms from brain homogenates infected with 22 L prions. Analyses of supernatants and pellets by western blot showed that MR100 is necessary to precipitate PrP isoforms. Indeed, if the RCA is performed with the solvent alone, precipitation does not occur and the rSDS-PrP^Sc^ oligomers are barely detected (Fig. 5b). Replacement of MR100 by P30 in the RCA protocol revealed that MR100 has a strong capacity to concentrate and induce rSDS-PrP^Sc^ oligomers, unlike the P30 compound. We tested this “rapid centrifugation assay” (RCA) using either uninfected murine brain homogenates or homogenates infected with the prion strain 22 L. In prion-infected brain homogenates, most of the PrP molecules were found in the pellet, including a substantial fraction of 50-75 kDa rSDS-PrP oligomers. By contrast, in control, uninfected brain homogenates (NBH), MR100 precipitated PrP^C^ but without detection of 50-75 kDa rSDS-PrP oligomers (Fig. 5c). These results indicate that MR100 oligomerizes prion-infected brain samples, but not uninfected ones. This property could be used to develop a new method to easily discriminate infected and uninfected brain samples without the PK digestion step, which is replaced by a short centrifugation step. Suppression of PK digestion could be an advantage as it allows the detection of all forms of infectious prions, particularly the sPrP^Sc^ forms [5–8]. We then used the RCA method in hamster brain tissues either uninfected or infected by the 263 K prion strain, and collected at the terminal stage of the diseases (Fig. 6a). No rSDS-PrP^C^ oligomers could be detected in normal hamster brain samples by RCA method, whereas rSDS-PrP^Sc^ oligomers were observed in prion-infected homogenates, with a strong depletion of PrPs proteins in the supernatant. We then used the RCA method in hamster brain tissues collected at various stages of disease (i.e., 109, 130 and 148 days after infection with the 263 K strain) (Fig. 6b). Immunoblot analyses after RCA showed the presence of substantial amounts of rSDS-PrP^Sc^ oligomers at day 130 and 148 (terminal stage of disease) and traces at day 109 post-infection. In order to compare the RCA with the method that includes the PK digestion step, aliquots of the same samples were incubated with MR100 for 1 h and then digested with PK at 37 °C for 1 h (Fig. 6c). In this case, only small numbers of oligomers were observed at day 148 and traces at day 130 post-infection, indicating that oligomers are very sensitive to PK treatment. No signal was detected at day 109 post-infection, suggesting that at this stage all PrP^Sc^ species are PK-sensitive (Fig. 6b and c). Finally, while testing the RCA, we noticed that better quality results were obtained when freshly homogenized brain tissues were used, possibly because successive freeze-thaw cycles favor the detection of nonspecific oligomers due to the appearance of protein aggregates.

### RCA can differentiate between brain samples from patients with vCJD and controls

**Fig. 7:**
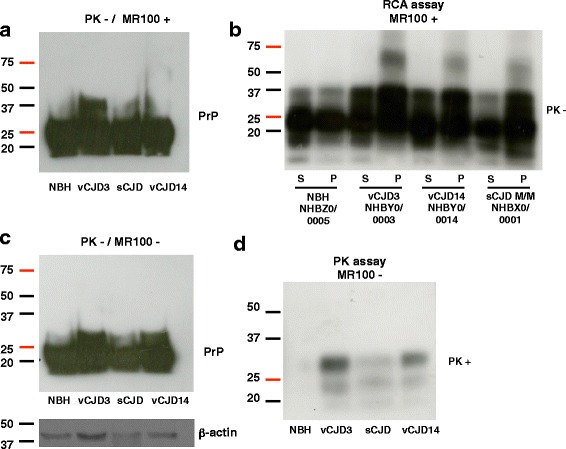
rSDS-PrP^Sc^ oligomers are detected in patients with new variant CJD (vCJD) when tested with RCA. **a-b** Frozen, homogenized brain samples from two patients with vCJD, one patient with sCJD (codon 129 M/M genotype) (positive control) and one healthy control (NBH) were from NIBSC. Each sample was identified by the number attributed by the NIBSC. The RCA assay was carried out as before (see legend to Fig. 5) but adapted to human samples: 50 μL of 10 % brain homogenates in 100 μL PBS/2 % Sarkosyl were incubated with 2 mM MR100 for 2 h. Before centrifugation, 30 μL was collected and mixed with 30 μL of 2X loading buffer for immunoblotting analysis (**a**). The rest of the sample was centrifuged at 11,000 g for 5 min, and supernatants (S) and pellets (P) were immunoblotted with the SAFmix (**b**) [16]. **c-d** For comparison, the same brain homogenates (50 μL of 10 % brain homogenates in 100 μL PBS/2 % Sarkosyl) were processed with the classical proteinase K digestion assay without MR100. Samples were analyzed before (**c**), and after proteinase K digestion (125 μg/mL PK at 37 °C for 1 h) (**d**). The reaction was stopped by addition of a protease inhibitor cocktail, before analysis of rPrP^Sc^ by western blotting with the SAF mix. Molecular masses (20–75 kDa) are on the left side of the panels

We tested whether RCA was suitable for human prion strains. Two homogenized brain samples (vCJD3 and vCJD14) were kindly provided by Dr Cooper (NIBSC, UK) together with a brain sample from a patient with sCJD (codon 129 Met/Met; sCJD M/M) and a normal brain homogenate (NBH, negative control) (Fig. 7). We first performed western blot analyses before proteinase K digestion and did not observe rSDS-PrP^Sc^ oligomers when we incubated the samples in presence or absence of the MR100 drug (Fig. 7a and c). This result suggested that these rSDS-oligomeric species either do not exist in this strain, or are too diluted in the original sample to be detectable by western blot. When we applied the RCA protocol developed on the rodent strain in the human samples, we again could not see the rSDS-oligomeric species. Therefore, we altered the experimental conditions to adapt the RCA protocol to prion-infected human samples. Briefly, more concentrated brain homogenates were used, the MR100 concentration was increased from 1.5 to 2 mM and the duration of the incubation with MR100 at room temperature from 1 to 2 h. Western blot analysis probed with the SAFmix revealed the presence of rSDS-PrP^Sc^ oligomers in the pellet of all CJD brain homogenates but not in the NBH sample (Fig. 7b). The centrifugation step combined with a slightly different RCA protocol allowed the concentration of these rSDS-PrP^Sc^ oligomers in the pellet and their detection by western blot. Thus, it seems that these species in human brain tissues are not as abundant as in rodent models because we could not visualize them prior to PK digestion. Because the marker PrP^Sc^ from vCJD strain has been well described for its lower resistance to PK digestion we analyzed these brain homogenates by PK digestion to compare the classical procedure to result obtained in the RCA (Fig. 7d). Immunoblot analysis showed the typical presence of rPrP^Sc^ in the infected samples with bands between 19 kDa and 33 kDa. The intensity of the signal after PK digestion was low, but in accordance with the western blot data posted on the NIBSC website (*http://www.nibsc.org/science_and_research/virology/cjd_resource_centre/available_samples/who_reference_reagents/nhby0_-_0003/western_blotting.aspx*).

### RCA reveals a correlation between the levels of rSDS-PrP^Sc^ oligomers in brain samples from patients with sCJD and the duration of the symptomatic phase of the disease

**Fig. 8:**
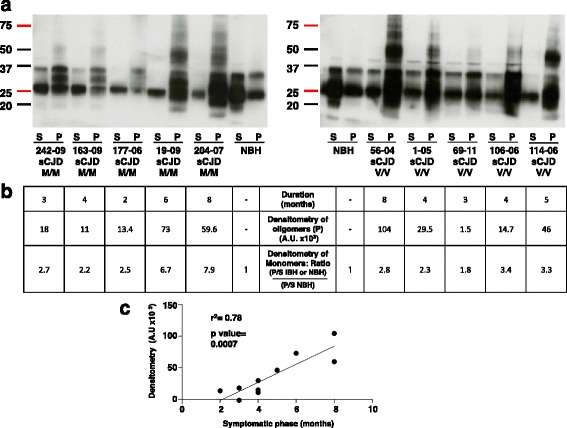
RCA efficiently detects rSDS-PrP^Sc^ oligomers in patients with sporadic Creutzfeldt-Jakob disease (sCJD). **a** Freshly homogenized human brain tissues from patients with sCJD (codon 129 Met/Met or Val/Val polymorphisms) were analyzed using RCA. The number on the figures corresponds to the identification numbers attributed by the tissue bank. The brain homogenate from a healthy control (NBH, the same as in Fig. 7) was used as negative control. 50 μL of 10 % human brain homogenates in 100 μL PBS/2 % Sarkosyl were incubated with 2 mM MR100 at room temperature for 2 h and centrifuged at 11,000 g for 5 min. Supernatants (S) and pellets (P) were immunoblotted with the SAF mix. Molecular masses (20–75 kDa) are indicated on the left side of the panels. **b** The table below shows: (*i*) the duration (in months) of the symptomatic phase of the disease; (*ii*) the results of the densitometry analysis of oligomers after subtraction of the background to normalize each value (ImageJ software); and (*iii*) the results of the densitometry analysis of the monomers in the pellet (P) and in the supernatant (S), and the calculated ratio (P/S) obtained for each sample (Additional file 1: Table S1 for calculation of ratios). **c** The correlation between the oligomer amount and the duration of the symptomatic phase of the disease in the 10 sCJD samples was assessed with the Pearson’s test using GraphPad (San Diego, CA, USA)

We processed ten frozen brain samples from patients with sCJD (five with the codon 129 Met/Met polymorphism and five with the codon 129 Val/Val polymorphism) using the adapted RCA protocol for human tissues. Most of the infected samples showed the presence of rSDS-PrP^Sc^ oligomers, but not the NBH control, and the intensity of the oligomer signal appeared to be proportional to the length of the clinical phase of the disease (Fig. 8a). Notably, in one sample sCJD177-06, which came from a patient presenting a short duration of the symptomatic phase of the disease, the rSDS-PrP^Sc^ oligomers were barely detectable. However, the pattern of precipitated monomers (the level of PrP monomers in the pellet compared to the amount of PrP monomers remaining in the supernatant) for the sample 177-06 is clearly distinguishable from the NBH. For each sample, we performed a densitometry analysis of the monomers in the pellet (P) and in the supernatant (S) and calculated the ratio P/S (see Additional file 1: Table S1). For normal brain homogenate (NBH) the ratio is 0.5, meaning that we have 2 times more PrP in the supernatant than in the pellet, whereas for the sample 177-06, the ratio is 1.28, meaning that we have more PrPs in the pellet than in the supernatant. We then normalized these values by dividing them by the ratio obtained by the non-infected sample NBH. Thus, for the NBH, the precipitation ratio of monomers is: 1 (0.5/0.5), whereas for 177-06 the value is 2.5 (1.28/0.5). The calculated ratio ranges were 2.2-7.9 for the samples from patients with the Met/Met polymorphism and 1.8-3.4 for the samples from patients with the Val/Val polymorphism (Fig. 8b). The ratios of monomers were all superior to 1 with the samples coming from sCJD patients. In addition, we noticed that the intensity of the oligomer signal seemed to be proportional to the length of the clinical phase of the disease, thus we performed a densitometry analysis of the oligomers for each sample (Fig. 8b). We used Pearson’s test with a probability of 0.05 (GraphPad software) and confirmed the presence of a significant correlation between duration of the symptomatic phase of the disease and intensity of the signal when all the ten sCJD samples were analyzed simultaneously (r^2^ = 0.78, p-value = 0.0007) (Fig. 8c).

Thus, MR100 results allow correlation of the amount of the rSDS-PrP^Sc^ oligomers with the progression of the pathology in sCJD samples. Altogether, our data suggest using both criteria, ratio of monomers and densitometry of rSDS-PrP^Sc^ oligomers to discriminate infectious samples from normal non-infectious samples.

### Pre-incubation of prion-infected brain inocula with MR100 greatly increases animals’ survival

**Fig. 9:**
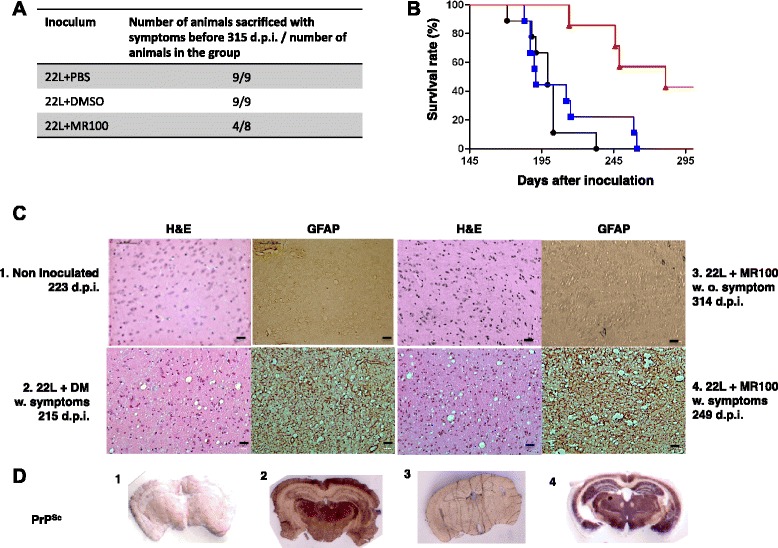
Pre-incubation of brain inoculum with MR100 substantially increases the survival time of injected animals. **a** Table with the number of animals sacrificed with symptoms before 315 days post-inoculation compared to the total number of animals for each group. **b** Kaplan-Meier survival plots of mice inoculated (15 μL/each mouse) with a 22 L-infected brain homogenate that was previously diluted in 2 % Sarkosyl/PBS and incubated at a final concentration of 1.5 mM MR100 (n = 8; 22 L + MR100; red) or an equivalent volume of DMSO (150 μL) (n = 10; 22 L + DM; blue) or PBS (150 μL) (n = 10; 22 L+ PBS; black) at room temperature for 2 h. **c** Histological analyses of thalamus (Th) sections from mice not inoculated with prions (1), or that were inoculated with 22 L + DM inoculum (2) or 22 L + MR100 inoculum and presented no symptoms (3) or with symptoms (4) of prion disease when they were killed (d.p.i., days post-inoculation). Tissue sections were stained with hematoxylin and eosin (HE) to confirm the prion pathology as indicated by the presence of vacuoles and probed with anti-GFAP antibodies as a marker of astrocytic gliosis. Tissue labeling was performed on several animals (2-3 per group) and images are representative of the staining observed in each group. **d** PET-blot analysis of frontal tissue sections of mice non-inoculated with prions (1), or that were inoculated with 22 L + DM inoculum (2), or 22 L + MR100 inoculum and presented no symptoms (3) or with symptoms (4) of prion disease when they were killed. The SAF84 antibody was used to detect rPrP^Sc^ proteins and the Vectastain ABC-AmP Kit (Vector laboratories, USA) to reveal antibody binding. Scale bars, 10 μM, w: with, w.o.: without symptoms

Previous experiments performed with the P30 thienyl pyrimidine compound showed that it could slightly decrease prion infectivity in bioassay experiments and increase survival time (from 154 to 163 days) [16]. As MR100 had a much stronger rSDS-PrP^Sc^ oligomer-inducing activity in cells, we expected that MR100 would trap infectivity more efficiently than P30. To test this, we pre-incubated 22 L-infected brain homogenates with 1.5 mM MR100 (22 L + MR100), 150 μL PBS (22 L + PBS) or 150 μL DMSO (22 L + DM) for 2 h before injection via stereotaxy into the brains of healthy Swiss mice. Immunoblot analysis of 22 L+ MR100 inocula confirmed the presence of rSDS-PrP^Sc^ oligomers after MR100 treatment (data not shown). Survival time of mice inoculated with 22 L + MR100 brain homogenates (n = 8; red) was substantially increased (non-parametric Mantel-Cox log-rank test, ** *p* = 0.007) compared to animals inoculated with 22 L + PBS (black) or 22 L + DM (blue) brain homogenates (n = 9 for both groups)(Fig. 9a-b). Remarkably, in the group of mice inoculated with 22 L + MR100 brain homogenates, 50 % of animals developed symptoms after an extended period of incubation, whereas the other 50 % survived without showing symptoms (Fig. 9a-b). The median survival time of animals with prion-disease symptoms was 281 days for mice injected with 22 L + MR100 brain homogenates, 199 days for the 22 L + PBS group and 191 days for the 22 L + DM group (Fig. 9b). High concentrations of MR100 can trap prion infectivity in the inoculum, increasing the animals’ survival time by about 30 % for the subgroup that develops the disease. Histopathological analyses were performed to compare the brain tissues of animals that were injected with 22 L + MR100 inocula and did or did not develop symptoms. Spongiosis and astrocytic gliosis (assessed by GFAP expression) were detected in the brain of mice with symptoms, but not in mice without symptoms that were sacrificed at 314 days post-inoculation (d.p.i.) or non-inoculated control animals (Fig. 9c). In addition, rPrP^Sc^ labeling was detected by PET-blot analysis (Fig. 9d) with the SAF84 antibody in the brain of sick animals injected either with 22 L + DM or 22 L + MR100 inocula and sacrificed with symptoms. However, no rPrP^Sc^ labeling was present in non-inoculated healthy control animals or mice inoculated with 22 L + MR100 killed at 314 d.p.i. without symptoms. Altogether, this data showed that MR100 can trap prions in the inoculum and is even more efficient than P30, because 50 % of animals were still alive and asymptomatic at 315 d.p.i. [16].

## Discussion

Due to the unique nature of prion agents and their mode of propagation, PrP^C^ and PrP^Sc^ (PrP27-30) are the main targets in therapeutic and diagnostic approaches. Most screening assays with prion-infected cells to identify new anti-prion drugs have focused on reducing the level of PrP(27-30). However, it is difficult to assess which PrP^Sc^ species (small PrP^Sc^ oligomers, large aggregates or amyloid fibrils) are targeted by a drug because, after PK digestion and in SDS and reducing conditions, all PrP^Sc^ species are dissociated, leading to a monomeric band known as PrP(27-30). In addition, global decrease of PrP^Sc^ levels in the cells is not always correlated with diminished infectivity [21]. A second strategy is based on the identification of small molecules that favor the detection of PrP(27-30) oligomers (also called rSDS-PrP^Sc^ oligomers) on western blot [16] as they could stabilize or cross-link PrP^Sc^ species [16]. Although it seems contradictory to promote the formation of such species, a recent study showed that a novel oligomer modulator (Anle138b) strongly reduced high molecular weight species and increased the survival time of prion and Parkinson’s disease mouse models [22]. Studies on Alzheimer’s disease have shown that small molecules can be powerful tools for the modulation of amyloid formation cascades and demonstrated that acceleration of fibril formation reduces Aβ_42_ toxicity in human neuroblastoma cells and in rat brain slices [23, 24]. Altogether these studies on oligomer modulators demonstrate that small molecules can redistribute the equilibrium between the various species of the amyloid cascade in prionopathies and that these strategies might be useful in identifying new compounds for diagnosis or therapeutic purposes.

Recently, we identified a family of thienyl pyrimidine compounds that favor rSDS-PrP^Sc^ oligomers and diminish prion infectivity when mixed with the prion-infected inoculum in bioassay studies [16]. In this work, we designed and synthesized an analog, MR100, with stronger rSDS-PrP^Sc^ oligomer-inducing activity to better understand its mechanism of action and further evaluate the diagnostic and therapeutic potentials of this family of molecules. MR100 is effective at nanomolar concentrations in N2a58/22 L cells and has a broad spectrum of action as indicated by its rSDS-PrP^Sc^ oligomer-inducing activity on rodent and human prion strains.

We used the fluorescence properties of MR100 to study the interaction of the molecule with two forms of the prion protein: either the soluble recPrP or the fibrillar PrP. MR100 is a hydrophobic compound, and in an aqueous environment its fluorescence is quenched. Upon binding with recPrP or PrP fibrils, the environment of MR100 is less aqueous and we observed an increase of the fluorescence of MR100 molecule (signal intensity doubled). Following incubation of MR100 with PrP fibrils, we also observed a strong red-shift of the emission spectrum from 452 nm to 460 nm, and a large shoulder appeared around 500 nm, illustrating substantial modifications in the environment of MR100. These differences suggest that MR100 may have different properties towards PrP isoforms. Indeed, SDS-PAGE gel experiments allowed us to directly visualize the binding of MR100 on hamster PrP fibrils due to the orange coloration of PrP bands with MR100, but also with the formation of rSDS-oligomer species. Altogether, these experiments indicate that MR100 compound interacts with large aggregates of PrP and is able to cross-link those species and form rSDS-oligomers in denaturing conditions. Remarkably, MR100 presents some structural analogies with a class of chemicals called the Luminescent Conjugated Oligothiophene (LCOs) that were recently shown to interact with a wide variety of amyloid fibrils and protein aggregates such as amyloid-beta and tau [25, 26]. Thus, MR100 may also interact with other types of amyloids *in vitro*.

We also evaluated the potential of MR100 as a diagnostic tool and we took advantage of the MR100 precipitation effect to develop the “rapid centrifugation assay” (RCA). This assay requires an incubation step with MR100 at room temperature, followed by a quick centrifugation, without need for PK digestion. We show that RCA can be used to differentiate 22 L- or 263 K-infected from healthy rodent brain samples by detecting the presence of 50-75 kDa PrP^Sc^ oligomers in the pellet fraction. Moreover, RCA allows the detection of traces of oligomers in hamster brain samples as early as day 109 post-infection, whereas following PK digestion no rPrP^Sc^ signal is visible until a later stage. Altogether, these data suggest that RCA could be used for the early detection of prions in animal models.

Finally, we adapted the RCA method to detect PrP^Sc^ oligomers in human brain homogenates. We show that it can differentiate between samples from patients with sCJD or vCJD and samples from healthy controls. Moreover, in samples from sCJD patients, the levels of PrP^Sc^ oligomers in the pellet was positively correlated with the duration of the symptomatic phase of the disease (r^2^ = 0.78, *** *p value* = 0.0007). This is remarkable because it suggests a link between the amount of a prion biomarker and the duration of the symptomatic phase of the disease in the affected patient. In addition, the tissue samples from patients with sCJD were cerebellar, a brain area that was previously associated with variability in the detection of prions by immunohistochemistry (IHC) in comparison to the cortical regions (50-63 % of the cerebellum specimens were positive by IHC compared to cortex) [11]. All the cerebellum samples tested by RCA were found positive, suggesting that our assay is quite sensitive. It will now be important to test samples from other brain areas to better define the RCA sensitivity. Phosphotungstic acid precipitation (PTA) is another method based on PrP precipitation that allows for better detection of prions in samples [27]. However, in the PTA protocol the protease digestion step is needed to differentiate PrP^C^ from PrP^Sc^ isoforms, whereas with RCA protease digestion is not required, allowing for detection of both sensitive and resistant PrP^Sc^ species. Compared to the PTA, the RCA method using MR100 is based on the detection of rSDS-PrP^Sc^ oligomers, which is not the case for PTA, since oligomers are usually proteinase K digested. Similar to the PTA, we noticed that MR100 is able to precipitate PrP^C^ but without forming rSDS-dimers/trimers of PrP^C^ by opposition to PrP^Sc^, and this effect is highly efficient in rodent strains (Figs. 7b and 8a). Our * in vitro* fluorescence studies with MR100 suggested a difference in the interaction between PrP isoforms. Indeed, with MoPrP(23-230) we observed an increase in the fluorescence of MR100, suggesting an interaction. However, with PrP fibrils, the fluorescence of MR100 is shifted from 452 nm to 460 nm and a shoulder appears around 500 nm, showing a strong modification in the environment of MR100 and suggesting a possible interaction with quaternary structural elements of PrP fibrils. MR100 is a highly hydrophobic compound and one can imagine that MR100 interacts with hydrophobic structure elements of PrP^Sc^, and is able to cross-link fibrils (Fig. 2d).

Recently, a novel prion disease, without *PRNP* mutations, was described by Gambetti [5]. This new disease, named “variably protease-sensitive prionopathy” (VPSPr), which represents 2-3 % of all CJD cases, is characterized by the presence of an abnormal prion protein in the brain that is highly sensitive to proteinase K. Indeed, examination of brain tissues from 26 patients showed that the genotype at codon 129 (MM, MV or VV) influenced the PK-resistance of PrP^Sc^ [5–8]. In addition, VPSPr cases do not have mutations in the PRNP gene, thus they are difficult to detect with classical assays. It would be of particular interest to test brain tissues from patients with VPSPr using the RCA to see whether PK-sensitive oligomers can be detected.

Since MR100 is effective at nanomolar concentrations in N2a58/22 L cells and has a broad spectrum of action, as indicated by its rSDS-PrP^Sc^ oligomer-inducing activity on rodent and human prion strains, we asked whether this oligomeric activity induced by MR100 could have an impact on prion infectivity. Pre-incubation of 22 L prion-infected brain homogenates with an excess of MR100 before inoculation into mouse brains substantially increased the survival times of animals compared to controls (p = 0.007) and, 50 % of animals survived without succumbing to the disease. This result is in agreement with our previous bioassay experiments obtained with P30-treated inoculi showing a slight decrease of the infectivity in mice [16]. Here, we showed that MR100 is much more efficient than P30 at trapping prion infectivity, probably due to the substantial difference in their activity (ED_50_ is 17 μM for P30, and 1 nM for MR100) [16]. As MR100 exhibits a strong ability to precipitate PrP isoforms compared to P30, we hypothesized that an excess of MR100 could form a protective shell around PrP species, which then aggregate and partially inactivate the prion strain, blocking pathways of prion replication. A direct application of this property might be the use of MR100 as a surface prion decontaminant. Interestingly, a recent paper published by Herrmann et al., [28] showed that administration of polythiophenes (compound structurally similar to MR100) to the brain of prion-infected mice via osmotic minipumps, led to a survival extension of 80 % and demonstrated activity against both mouse and hamster prions. Due to the similarity in chemical structure between MR100 and these oligothiophenes, and because oligothiophenes have the ability to generate SDS-stable PrP^Sc^ oligomers such as for MR100, we expect that treatment with MR100 using a protocol with osmotic minipump may lead to an increase of the survival life of prion-infected animals.

## Conclusion

To conclude, MR100 is a promising tool for studying the mechanism of prion propagation and has allowed the development of an alternative method for the identification of prion-infected brain samples based on the rSDS-PrP^Sc^ oligomers that could be useful for specific purposes. We are now testing whether this assay can be adapted for biological fluids, especially blood. Regarding our bioassay results, we will further explore the potential of MR100 as a prion decontaminant.

## Methods

### Ethics statement

This project follows the specific French national guidelines on animal experimentation and animal well-being and was approved by the Ethic Committee for Animal Experimentation (“Effet des dérivés thiényls pyrimidiques sur le mécanisme de réplication des prions”, Nr. CE-LR-11001).

Tissue samples from individuals with CJD and controls were from: (*i*) the collection of the brain bank of the Institute of Neurology of the Medical University of Vienna. Samples were collected following local regulations for diagnostic purposes in the frame of CJD Surveillance. Anonymized tissue samples remaining after diagnosis were used in this project in the framework of the “Molecular neuropathologic investigation of neurodegenerative diseases” study that was approved by the Ethical Committee of the Medical University of Vienna (Nr. 396/2011), and followed the principles of the Helsinki declaration; (*ii*) the collection of the CJD Resource Center, National Institute for Biological Standards and Control (NIBSC, UK) covered by the National Research Ethics Service (NHS, UK) with the REC ref number 09/H0405/2. Samples provided by NIBSC were anonymized.

### Human samples

Brain samples **(**10 % (w/v) homogenate in 0.25 M sucrose/PBS) from patients with new variant CJD (vCDJ) (NHBY0/0003 and NHBY0/0014) were provided by the National Institute for Biological Standards and Control (NIBSC, UK, *www.nibsc.ac.uk*). A normal human brain sample (NHBZ0/0005) and a sample from a patient with sporadic CJD (sCDJ) (codon 129 Met/Met polymorphism) (NHBX0/0001) were also provided as controls. Frozen cerebellum specimens from five patients with sCJD of Type 1 (codon 129 Met/Met polymorphism) and from five patients with sCJD of Type 2 (codon 129 Val/Val polymorphism) were provided by Dr. Kovacs (Institute of Neurology, Vienna).

### Biological reagents and antibodies

Pefabloc and proteinase K were purchased from Roche Diagnostics (Mannheim, Germany). The protein assay kit based on the bicinchoninic acid (BCA) was purchased from Pierce (Thermofisher Scientific, Saint Herblain, France). For immunoblotting analyses, we used a mix (SAF mix) of three anti-PrP antibodies (SAF60, SAF69 and SAF70) that were kindly provided by Dr. Jacques Grassi (CEA, Saclay, France). The SAF69 antibody is critical for detection of PrP oligomers. The SAF84 antibody used for pet-blot analyses were purchased from SpiBio (Montigny-le-Bretonneux, France). Secondary antibodies were from Jackson ImmunoResearch (West Grove, PA, USA). All other chemicals and antibodies were purchased from Sigma (Paris, France).

### Chemical reagents

The thienyl pyrimidine compounds A6, A12, A13, A14, A17 and A18 that have rSDS-PrP^Sc^ oligomer-inducing activity [16] were purchased from Maybridge (Cornwall, United Kingdom) and Key Organics Limited (Cornwall, United Kingdom). Stock solutions were prepared at 5 mM and drugs were solubilized in DMSO according to the suppliers’ recommendations. For the synthesis of MR1 (6-(thiophen-2-yl)-1,3,5-triazine-2,4-diamine), MR2 (6-(5-bromothiophen-2-yl)-1,3,5-triazine-2,4-diamine), MR3 (5,5'-bistrimethyltin-2,2'-bithiophene) and MR100 (6,6'-(2,2′:5′,2′′:5′′,2′′′-quaterthiophene-5,5'''-diyl)bis(1,3,5-triazine-2,4-diamine)), reactions were carried out using dried or freshly distilled solvents, under a dry argon atmosphere and according to the Stille cross-coupling pathway described recently [18]. Elemental analyses were performed by the "Service Central d’Analyse du CNRS (Solaize, France)". ^1^H NMR spectra were recorded at 300.75 MHz, and ^13^C{^1^H} NMR spectra at 75.30 MHz using a Bruker AV-300 instrument. Chemical shifts (δ) were related to the residual solvent peak as internal standard. δ and coupling constant values (J) were expressed in ppm and Hz, respectively.

### Cell culture

The mouse neuroblastoma cell line N2a was purchased from the American Type Culture Collection (ATCC CCL131). The N2a58 subclone, which over-expresses mouse PrP (MoPrP), was chronically infected with the mouse-adapted scrapie strain 22 L (N2a58/22 L cells), as described by Nishida [29]. N2a58 and N2a58/22 L cells were cultured as described previously [16].

### Cell screening assay, dose-response curves and immunoblotting

Drug screening using N2a58 or prion-infected N2a58/22 L cells was performed as previously described [16]. Briefly, 10^6^ cells/25-cm^2^ flasks were incubated with 20 μM of each compound (final concentration) for 4 days. At confluence, cells were lysed in 400 μl lysis buffer (0.5 % NP-40, 0.5 % Deoxycholate, 10 mM Tris-HCl pH 8, 100 mM NaCl) and protein concentration was measured using the BCA assay. For western blots, all samples were normalized regarding their protein amount and volumes. For the dose-response curve (Fig. 1d), aliquots of 20 μL were taken as protein loading controls probed with glyceraldehyde-3-P dehydrogenase (G3PDH) or β-actin. Then, normalized samples were digested with 20 μg/ml PK at a ratio of 1:25 (protease to protein) at 37 °C for 1 h. Digestion was stopped with 1 mM Pefabloc and samples were centrifuged at 20 000 g at 4 °C for 30 min. Pellets were dissolved in 20 μl lysis buffer and 20 μl 2x loading buffer (0.1 M DTT, 3 % SDS, 20 % glycerol, 0.4 M Tris-HCl pH 7.4 and bromophenol blue), then boiled for 3 min before loading on 12 % SDS-PAGE Criterion precast gels (Biorad). Western blotting was performed according to standard procedures and MoPrP was detected with the SAF mix, as previously described [30]. For the dose-response curves, about 1×10^6^ N2a58/22 L cells were plated in 25-cm^2^ flasks and incubated with P30, A6 or MR100 at various concentrations (from pM to μM) for 4 days. At confluence, cells were lysed, extracts were normalized for protein amounts and PK-digested as described above. Samples were analyzed by western blotting using the SAF mix, as described previously [30].

### Purification of prion protein and formation of amyloid fibrils

Full-length recombinant mouse PrP encompassing residues 23-230 (MoPrP23-230) was expressed in *E. coli* and purified as described previously [31]. The purified recombinant MoPrP23-230 was confirmed by SDS-PAGE and electrospray mass spectrometry to be a single species with an intact disulfide bond and correct molecular weight as previously described in Ayrolles-Torro et al., [16]. After purification, MoPrP was stored in lyophilized form at -20 °C until use.

Amyloid fibrils using full-length MoPrP23-230 were formed using the manual setup protocol of Breydo et al. [32], and fibril formation was monitored by collecting aliquots to which 10 μM of thioflavin T were added as described previously. The quality of freshly made fibrils was also confirmed by transmission electron microscopy as described previously [33]. Two amyloid strains: hamster S-fibrils (for “shaking”) and hamster R-fibrils (for “rotation”) were kindly provided by Dr. Ilia Baskakov. Hamster fibrils were produced *in vitro* using full-length hamster PrP, according to the manual format protocol as described by Makarava and Baskakov [19].

### Absorption spectroscopy of MR100 compounds and fluorescence interaction studies

Five millimolar stock solution of MR100 compound was diluted at a final concentration of 5 μM, in 50 mM MES buffer pH 5, 1 % DMSO. Absorption spectra were recorded from 200-600 nm using a spectrophotometer Specord 250 (Analytikjena, France) and MR100 compound exhibits a maximal absorption wavelength at 470 nm (λmax). This wavelength (λmax = 470 nm) was selected for excitation of MR100 molecule using a fluorimeter FluoroMax2 (JobinYvon Spex, Tokyo, Japan). Lyophylized MoPrP(23-230) was solubilized at 0.5 mg/mL (0.2 mM) in 50 mM MES buffer pH 6, and filtrated on 0.2 μm filter. Then, Mo(PrP23-230) stock solution was diluted in 50 mM MES buffer at a final protein concentration of 4.4 μM, and mixed with 50 μM of MR100 compound, 1 % DMSO, during 2 h at 25 °C. Fluorescence experiments were conducted by excitation of the MR100 compound and emission spectra were recorded by excitating at 470 nm. Alternatively, MoPrP(23-230) at 0.5 mg/mL was mixed with 50 μM of MR100 (diluted in 50 mM MES buffer pH 5, 1 % DMSO) and incubated for 2 h at 37 °C. Fluorescence analyses of tryptophan and tyrosine residues of PrP were performed by excitation of samples at 295 nm. Emission spectra between 300 and 400 nm were recorded by exciting the fluorescence at 295 nm before the incubation of PrP with MR100 (T0) and after 2 h of incubation (T120).

### SDS-PAGE gel electrophoresis interaction studies

Hamster S- or R-fibrils (4.4 μM) were incubated with 40 μM of P30, A6 or MR100 compounds diluted in 50 mM MES buffer, 1 % DMSO, during 2 h at room temperature. Samples were mixed with 4X Loading buffer, boiled 15 min at 90 °C, and loaded on SDS-PAGE gel 12 %. Proteins in gel were revealed by silver staining.

### Aggregation assay using brain homogenates

Brain tissues from terminally sick mice (infected with 22 L prions) and hamsters at various stages of disease (infected with the 263 K strain) were homogenized in 10 % (w/v) PBS using microbead-containing tubes and a Ribolysor apparatus (Biorad, Marnes la Coquette, France). Samples were shaken for 45 s and then homogenates were collected with an insulin syringe to obtain a homogeneous suspension. Protein concentration was measured using the BCA assay and samples were normalized in order to get equivalent amounts of proteins and volumes. Before incubation of samples with compound MR100 and/or proteinase K, 20 μL of the normalized aliquots were collected as loading controls probed with β-actin.

To determine MR100 oligomer-inducing activity in brain homogenates, 50 μL of infected brain extracts (10 % w/v) were diluted in PBS/2 % Sarkosyl and incubated with 1 mM MR100 or DMSO (control) in a final volume of 0.5 mL for 1 h. Rodent samples were then PK-digested according to the previously described protocol [16]. For human samples, various PK concentrations were tested and an appropriate signal on western blot was obtained after digestion with 125 μg/ml PK at 37 °C for 1 h. Fifty microliters of each sample were mixed with an equal volume of 2x loading buffer and boiled for 3 min. Thirty microliters were loaded onto 12 % SDS-PAGE gels and analyzed by western blotting as described above using the SAF mix.

### Rapid Centrifugation Assay (RCA)

Fifty microliters of 10 % (w/v) rodent brain homogenates (22 L- or 263 K-infected and non-infected) were diluted in 300 μL 2 % Sarkosyl/PBS and incubated with 150 μL DMSO or P30 or MR100 at a final concentration of 1.5 mM at room temperature for 1 h. Samples were then centrifuged in a benchtop centrifuge (Eppendorf) at 8000 g for 5 min. Supernatants were removed and 30 μL aliquots were mixed with an equal volume of 2x loading buffer. Pellets were suspended in 30 μL 2 % Sarkosyl/PBS and mixed with an equal volume of 2x loading buffer. Supernatants and pellets were analyzed by western blotting using the SAF mix. For human samples (vCJD, sCJD and negative controls), 50 μL of 10 % (w/v) brain homogenates were diluted in 100 μL 2 % Sarkosyl/PBS and incubated with 100 μL MR100 at a final concentration of 2 mM at room temperature for 2 h. Samples were then centrifuged in a benchtop centrifuge (Eppendorf) at 11 000 g for 5 min. Pellets and supernatants were then processed like for the rodent samples.

### Bioassay using 22 L-infected brain homogenates treated with MR100

We incubated 50 μL of a 10 % solution (w/v) of 22 L-infected brain homogenates in PBS with 2 % Sarkosyl (300 μL) and 1.5 mM MR100 (150 μL) or 150 μL DMSO (DM) or PBS as controls, at 20 °C for 2 h. To determine prion infectivity, samples were diluted (10^5^ times) with normal brain homogenates before intracerebral inoculation as described previously [16]. Fifteen microliters of MR100-treated (n = 8), DMSO-treated (n = 10) or PBS-treated samples (n = 10) were injected into the lateral ventricle of Swiss mice using a stereotaxic frame (Kopf Instruments, Tujunga, CA, USA) with coordinates as follows: L: 1.0 mm; R: 0 mm; and P: -3.0 mm [34]. Groups of five mice were housed in cages placed in a ventilated protective cabinet. Mice were scored positive for prion disease when three signs of neurologic dysfunction were observed and when progressive deterioration (according to 16 diagnostic criteria) was apparent, as described previously [35, 36]. Once clinical signs were detected, animals were observed daily and killed *in extremis.* Their brain was removed and immediately frozen at –80 °C or fixed in AntigenFix (Diapath, France) for immunohistochemical analysis.

### Immunohistochemistry

Brain tissues were fixed in AntigenFix solution (Diapath, France) for 24 h. Then, they were decontaminated for 30 min in formic acid solution according to the protocol described by Andréoletti et al. [37] and stored in 100 mM phosphate buffer at pH 7.4 with 0.02 % sodium azide. Samples were dehydrated in graded ethanol, cleared in cedar oil and embedded in paraffin. Frontal 6- to 10-μm sections were cut using a microtome and mounted on Superfrost Plus slides (Microm France, Francheville). Sections were dewaxed and stained with hematoxylin and eosin as described previously [38]. Immunolabeling with anti-GFAP antibodies was performed according to the instructions provided with the Strept ABC Complex Kit. Labeling was visualized using 3-3’-diaminobenzidine chromogen solution (Sigma, France). For paraffin-embedded tissue blots (PET-blots), 6 μm frontal sections were cut using a microtome and placed on nitrocellulose membrane. After drying at 50 °C for 48 h, sections were dewaxed, digested with 25 μg/mL PK at 56 °C overnight and then denatured with 3 M guanidine thiocyanate for 10 min. Membranes were blocked with casein for 30 min. The SAF84 antibody was used to label rPrP^Sc^ and the Vectastain ABC-AmP kit (Vector laboratories, USA) to reveal antibody binding.

### Software and statistical analysis

ImageJ (http://rsb.info.nih.gov/ij/index.html) was used to compare the intensity of individual western blot bands. It uses a graphical method that generates lane profile plots by drawing lines to enclose the peaks of interest and then measuring the peak area using the wand tool. For each band, we obtained values corresponding to the defined area.

Correlations between the duration of the disease symptomatic phase and the oligomer amounts were calculated using the GraphPad software (San Diego, CA, USA) and Pearson’s test. Correlations were considered significant at p ≤ 0.05. Kaplan-Meier survival curves were created using the GraphPad software and the difference between curves was tested using the non-parametric Mantel-Cox test, with a probability of 0.05 defined as significant difference.

## Additional file

Additional file 1: Table S1. Densitometry analysis of monomers in the pellet (P) and in the supernatant (S) fractions to determine ratios (P/S). (PDF 54 kb)

## Abbreviations

CJD: Creutzfeldt-Jakob Diseases
DMSO: Diméthylsulfoxyde
IBH: Infected brain homogenate
IHC: Immunohistochemistry
MoPrP: Mouse prion protein
NBH: Normal brain homogenate
PK: Proteinase K
PrP: Prion protein
PrP^C^: Cellular prion protein
PrP^Sc^: Scrapie prion protein
PTA: Phosphotungstic acid precipitation
RCA: Rapid centrifugation assay
recPrP: recombinant PrP
rSDS: SDS-resistant
SAF: Scrapie-associated fibrils
sCJD: sporadic CJD
SDS: Sodium dodecyl sulfate
sPrP^Sc^: sensitive PrP^Sc^
vCJD: new variant CJD
VPSPr: Variably protease-sensitive prionopathy
