# Supplementary material for: A Fluorescent Oligothiophene-Bis-Triazine ligand interacts with PrP fibrils and detects SDS-resistant oligomers in human prion diseases
Source: Mol Neurodegener. 2016 Jan 26;11:11. doi: 10.1186/s13024-016-0074-7 (PMC4727337; doi:10.1186/s13024-016-0074-7)
Supplement: Additional file 1: Table S1. — Densitometry analysis of monomers in the pellet (P) and in the supernatant (S) fractions to determine ratios (P/S). (PDF 54 kb) [file 13024_2016_74_MOESM1_ESM.pdf]

Supplementary Table 1 :

**Samples sCJD**	**Densitometry of monomers in the Supernatant (S)**	**Densitometry of Monomers in the Pellet (P)**	**Ratio P/S**	**Normalized ratio (P/S Sample) / (P/S NBH)**
NBH	113041	58444	0.5	1
242.09	65572	90975	1.38	2.7
163.09	45815	52286	1.14	2.2
177.06	27868	35704	1.28	2.5
19.09	31932	108068	3.38	6.7
204.07	34678	137585	3.96	7.9
NBH	119361	81661	0.68	1
56.04	76019	146129	1.92	2.8
1.05	80148	127821	1.59	2.3
69.11	73939	93777	1.26	1.8
106.06	59289	139896	2.35	3.4
114.06	50790	116196	2.28	3.3
